# Integrated Approach in Systems Biology

**DOI:** 10.1155/2014/656473

**Published:** 2014-12-31

**Authors:** Huiru Zheng, Rui Jiang, Zhongming Zhao

**Affiliations:** ^1^Computer Science Research Institute, School of Computing and Mathematics, University of Ulster, Shore Road, Newtownabbey, County Antrim BT37 0QB, UK; ^2^Bioinformatics Division, Tsinghua National Laboratory for Information Science and Technology, Department of Automation, Tsinghua University, Beijing 100084, China; ^3^Departments of Biomedical Informatics, Psychiatry, and Cancer Biology, Vanderbilt University School of Medicine, Nashville, TN 37232, USA

Systems biology is a field within biology, aiming at understanding biological processes at the systems level and emerging from dynamic interactions of individual components that operate at multiple spatiotemporal scales. It is now an established and fundamental interdisciplinary research field. Systems biology studies biological systems by systematically perturbing them (biologically, genetically, chemically, or other); monitoring the gene, protein, metabolite, and informational pathway responses; integrating these data; ultimately formulating mathematical models that describe the structure of the system and predict its response to individual perturbations. Integrated “omics” (such as genome-wide measurements of transcripts, protein levels, or metabolite level) approaches have created exciting opportunities for systems biology and other biological researches thanks to the rapid advancement of high throughput biotechnologies. Computational methods such as data preprocessing, representation, modeling, measurement, interpretation, prediction, visualisation, and simulation have been well applied to understand biological processes and biological systems.

We organised this special issue to provide an international forum to discuss the most recent developments in the field regarding integrated data analysis approaches in systems biology research such as pattern recognition and prediction, modeling and simulation, and data representation and visualisation. This special issue featured “integrated approach” and “complex biological system” themes. We are interested in both new theories and tools in this area as well as their applications in systems biology. The potential topics include (i) large-scale or cross-species data integration for the reconstruction of networks and pathways; (ii) genomic data analysis using systems biology approaches; (iii) quantitative understanding of the dynamics of regulatory, signaling, interaction, and metabolic networks through modeling and simulation techniques; (iv) prediction of protein/RNA structure and biological network-based interactions; (v) data integration and knowledge-driven approach in biomarker identification and drug discovery; (vi) enhancement and enablement of knowledge discovery in functional genomics of disease and other phenotypes through integrated omics approach; (vii) semantic webs and ontology-driven biological data integration methods; (viii) development of integrated systems biology visualisation and analysis tools; (ix) development of integrated systems biology visualisation and analysis tools; (x) integrating approaches in transitional bioinformatics and personalized medicine.

Eight manuscripts were submitted in response to this special issue and six were finally accepted for publication, ranging from mathematical model, computational pipeline, and engineering design to computational models, with the applications at molecular, neuronal, protein, gene regulatory network, and gene ontology levels.

The comparison on biology sequences is one of the most important tasks in analyzing similarities of function and properties. W. Deng and Y. Luan integrated the dual-vector curve (DV-curve) and the detailed hydrophobic-hydrophilic (HP) model of amino acids, in the representation and visualization of protein sequences. Although the information might be lost in the representation, their results showed that the proposed method is efficient and feasible when focusing on the important part of the sequences.

L. Pasotti and S. Zucca reviewed the recent advances and computational tools in biological engineering design on which predictability issues in promoters, ribosome binding sites, coding sequences, transcriptional terminators, and plasmids were specifically discussed. The authors suggested that bottom-up approaches are urgently needed in order to refine and exploit the full potential of synthetic biology and a mixture of prediction tools could rapidly boost the efficiency of biological engineering by providing a smaller search space than fully random-based approaches.

In “*A pipeline for neuron reconstruction based on spatial sliding volume filter seeding*,” D. Sui et al. proposed a pipeline with a new seeding method for the construction of neuron structures from three-dimensional microscopy images stacks, which will be beneficial to three- dimensional neuron reconstruction and detection.

Gene regulatory networks consist of interactions between large number of genes and their regulators and are involved in every biological process. L. P. Tian et al. designed a state observer to estimate the states of genetic regulatory networks with time delays from available measurements. Furthermore, based on linear matrix inequality approach, a gene repressillatory network was employed to illustrate the effectiveness of the proposed design approach.

In “*Effects of maximal sodium and potassium conductance on the stability of Hodgkin-Huxley model*,” Y. Zhang et al. applied stability theory in the model design to investigate the importance of maximal sodium conductance and maximal potassium conductance. The study could help in researches relevant to diseases caused by maximal conductance anomaly.

In “*Correlating information contents of gene ontology terms to infer semantic similarity of gene products*,” the author proposed a new semantic gene ontology similarity measurement. A gene product was represented as a vector that is composed of information contents of gene ontology terms annotated for the gene product, and the pairwise similarity between two gene products was viewed as the relatedness of their corresponding vectors using three measures: Pearson's correlation coefficient, cosine similarity, and Jaccard index.

